# Adolescent girls’ descriptions of dysmenorrhea and barriers to dysmenorrhea management in Moshi, Tanzania: A qualitative study

**DOI:** 10.1371/journal.pgph.0001544

**Published:** 2023-07-06

**Authors:** Emily M. Cherenack, Jennifer Rubli, Abraham Melara, Nada Ezaldein, Aisha King, Maria L. Alcaide, Patricia Raccamarich, Lydia A. Fein, Kathleen J. Sikkema

**Affiliations:** 1 Department of Public Health Sciences, University of Miami, Miami, Florida, United States of America; 2 Department of Psychology and Neuroscience, Duke Global Health Institute, Duke University, Durham, North Carolina, United States of America; 3 Femme International, Moshi, Tanzania; 4 Division of Infectious Diseases, Department of Medicine, University of Miami, Miami, Florida, United States of America; 5 Graduate School of Public Health and Health Policy, City University of New York, New York, New York, United States of America; 6 Department of Obstetrics, Gynecology, and Reproductive Sciences, University of Miami, Miami, Florida, United States of America; 7 Department of Sociomedical Sciences, Columbia University, New York, New York, United States of America; University of Canberra, AUSTRALIA

## Abstract

Dysmenorrhea (menstrual pain) is common among adolescent girls globally, but many girls in Sub-Saharan Africa do not receive effective treatment. Qualitative interviews were used to describe adolescent girls’ experiences of dysmenorrhea and identify sociocultural barriers to dysmenorrhea management in Moshi, Tanzania. From August to November 2018, in-depth interviews were conducted with 10 adolescent girls and 10 adult experts (e.g., teachers, medical providers) who have experience working with girls in Tanzania. Thematic content analysis identified themes related to dysmenorrhea, including descriptions of dysmenorrhea and the impact of dysmenorrhea on well-being, as well as factors influencing the use of pharmacological and behavioral pain management strategies. Potential barriers to dysmenorrhea management were identified. Dysmenorrhea negatively impacted the physical and psychological well-being of girls and hindered girls’ ability to participate in school, work, and social events. The most common pain management strategies were resting, drinking hot water, engaging in physical activity, and taking paracetamol. Barriers to dysmenorrhea management included beliefs that medications are harmful to the body or can hinder fertility, limited knowledge about the benefits of hormonal contraceptives to manage menstruation, little continuing education for healthcare providers, and a lack of consistent access to effective medications, medical care, or other supplies necessary for pain management. Medication hesitancy and inconsistent access to effective medication and other menstrual supplies must be addressed to improve girls’ ability to manage dysmenorrhea in Tanzania.

## Introduction

Dysmenorrhea, defined as abdominal cramping and pain associated with menstruation, is a significant public health concern for adolescent girls around the world [1, 2]. In Sub-Saharan Africa (SSA), between 61%-84% of adolescent girls experience dysmenorrhea, with severe pain occurring among 33%-56% of girls [3–8]. In two studies from the Tanzania (the location of the current study), 75% of adolescent girls reported dysmenorrhea and 42% of girls reported very strong pain [7, 8]. In both SSA generally and in Tanzania specifically, dysmenorrhea has been shown to disrupt participation in school and social events, with dysmenorrhea linked to difficulty paying attention in class, school absenteeism, decreased school performance, difficulties engaging in physical activity, and an inability to socialize with friends [7–14]. Despite the high prevalence of dysmenorrhea in SSA, most adolescent girls do not receive first-line treatments for menstrual pain [7, 13, 15, 16]. Thus, understanding factors that influence the management of dysmenorrhea is critical to improving both sexual and reproductive health (SRH) and overall well-being among girls in SSA.

First-line treatments for primary dysmenorrhea include non-steroidal anti-inflammatory medications (NSAIDs) and hormonal contraceptive pills [2]. NSAIDs show especially high efficacy for primary dysmenorrhea because they are prostaglandin synthetase inhibitors [17, 18], and menstrual pain occurs in part due to the release of prostaglandins [1, 2]. Testing for gynecological and hormonal conditions is recommended among girls whose pain does not respond to NSAIDs and hormonal contraceptives [2]. However, these first-line treatments are not universally available or acceptable across settings [19].

In research from SSA, the proportion of women and girls seeking medical care for menstrual disorders ranges from 9%-16%, and many women and girls report “putting up with” pain [13, 15, 16]. Dysmenorrhea management strategies reported by girls in SSA have included resting, medication, herbal treatments, dietary changes, heat applied to the abdomen, exercise, and increased water intake [10, 11, 15]. Across five studies, the use of medications such as analgesics ranged from 43% to 64%, with 55% of girls in Tanzania with dysmenorrhea taking medications for menstrual pain [6, 7, 11, 13, 15]. In many cases, the non-NSAID analgesic paracetamol (i.e., acetaminophen), was the most common drug used, which is not a first-line treatment [6, 7, 11, 13, 15]. Few girls in SSA report using hormonal contraceptives to manage menstrual pain [7, 13, 15]. There is evidence that sociocultural factors may serve as barriers to accessing medications: in Uganda, adolescent girls reported limited access to pain medications and beliefs that medications can be harmful to the body [12].

Although prior research has described girls’ use of pain management strategies in SSA [10, 11, 15], there is still little insight into the psychosocial and cultural factors driving the management of dysmenorrhea in Tanzania. In Tanzania, difficulties managing dysmenorrhea may be heightened because menstruation is often stigmatized and girls can lack access to menstrual education, menstrual supplies, reproductive healthcare, and social support [7, 8, 20–27]. A health barrier is any factor that makes it more difficult for an individual to use health services (including prevention, diagnosis, or treatment) or improve their health in any other way [28]. To be more effective, interventions for dysmenorrhea should be culturally tailored to address barriers that impact girls’ ability to access menstrual care and manage dysmenorrhea. Therefore, this research analyzed qualitative interviews with adolescent girls and adult experts who work with adolescent girls (e.g., teachers, medical providers, parents, and non-governmental organization (NGO) staff) in Tanzania to describe 1) girls’ experiences of dysmenorrhea, 2) sociocultural factors impacting girls’ ability to manage dysmenorrhea, and 3) barriers to dysmenorrhea management.

## Materials and methods

### Setting and approach

From August 2018 to November 2018, qualitative semi-structured interviews were conducted as part of a larger study among adolescent girls in the Kilimanjaro region of Tanzania, which includes the municipality of Moshi. The larger study sought to characterize the types of stressors girls experience related to menstruation and puberty, how girls cope with stressors, and how stress and coping impact mental health and reproductive health. As interviews progressed, it became apparent that dysmenorrhea was a major stressor for girls, and additional questions were added to gain a deeper understanding of dysmenorrhea and menstrual pain management. The current analysis presents findings specific to dysmenorrhea.

### Researcher characteristics

The research team consisted of a principal investigator from the United States (EMC) who has a background in clinical health psychology, as well as research assistants and co-investigators from Tanzania and the United States. Paid research assistants from Tanzania (two young adult women) who had experience working with adolescent girls conducted all study activities with participants in Swahili. Coding was completed by the first author (EMC) and two undergraduate research assistants, and themes were discussed with research assistants in Tanzania. To acknowledge and reduce sources of bias, the team held ongoing discussions about reflexivity, bias, and positionality.

We employed a community-engaged approach to increase the relevancy of research questions, improve the validity of findings, enhance inclusivity in global research, and gain a better understanding of the cultural factors impacting girls’ experiences during puberty. As such, this study was implemented in partnership with Femme International, a local NGO that conducts menstrual needs assessments and provides menstrual education and supplies to adolescent girls, and Kilimanjaro Christian Medical University College, a regional research hospital. Partner organizations, members of the local community (e.g., staff at multiple NGOs, medical providers, religious leaders, community leaders), and a community advisory board of men and women hosted by Kilimanjaro Christian Medical University College were consulted prior to study design and throughout implementation. They provided input on research aims, methods, interpretation of outcomes, and dissemination of results. Findings were presented directly to the aforementioned community members and community advisory board, who agreed with the results.

### Sample

An *a priori* sample of 10 adolescent girls and 10 adult experts was selected based on prior research [29]. Based on the reoccurrence of similar themes, the study team determined that saturation had likely been reached by the end of recruitment. Adolescent girls were eligible if they were 14–19 years old and had reached menarche. We used non-random purposive sampling to recruit girls who varied in age, years in school, pregnancy history, marriage status, socioeconomic status, rural/urban region, and religion. Adult experts were defined as adults who worked or lived with adolescent girls. We aimed to recruit at least one teacher, healthcare provider, NGO staff member, local government leader, faith community leader, mother, and father. Tanzanian research assistants recruited participants through presentations and face-to-face recruitment in settings where adolescent girls are likely to be found. This included public markets, NGOs, schools, and churches/mosques. Adult experts were contacted via phone and in person based on employment settings (for example, pharmacies, schools, and shelters).

### Procedure

Ethical approval for human subjects research was obtained from the Duke University and Kilimanjaro Christian Medical University College Institutional Review Boards and the National Institutes of Medical Research in Tanzania. The Tanzania Commission for Science and Technology granted permission for the principal investigator to conduct this research. The Moshi Municipal Council provided permission to recruit in schools. Consent forms were translated into Swahili and reviewed by local NGO staff to ensure they were understandable to girls. All participants provided written and signed informed consent. A waiver of parental consent was requested for girls under 18 and granted by the regulatory agencies to protect girls’ privacy by allowing girls to participate without having to disclose their menstrual status to caregivers. This was essential because discussing menstruation with men is considered taboo, and having to disclose menstrual status to male caregivers could prevent girls from being represented in this research [20].

Interviews consisted of a single session lasting between 30 minutes and an hour. Interviewers took part in supervision after each interview and received training on interpersonal strategies to enhance the girls’ comfort. The larger theory of interest for the study was the transactional model of stress and coping, which sought to understand the link between stress, coping, and mental health among girls [30]. Semi-structured interview guides were designed to gather information on three main themes: (1) what are the challenges or stressors girls face relating to puberty and menstruation, (2) how are girls coping with or managing these challenges, and (3) how and why do stress and coping impact mental health? To avoid biasing the interviews to be overly focused on stressors, questions were also asked about positive experiences during menstruation and puberty.

As interviews progressed, it became apparent that dysmenorrhea was a significant stressor for girls, and additional questions were added to probe more deeply on characteristics of dysmenorrhea (e.g., pain location and severity), treatments used for dysmenorrhea, reasons for using specific treatments, and the effectiveness of treatments. Interviews with adult experts included additional questions on resources available to girls to manage dysmenorrhea and the role of adults (e.g., parents, teachers, medical providers, and community leaders) in helping girls manage menstrual stressors. Table 1 presents examples of actual questions asked by interviewers. Interviewers led with open-ended questions and followed up with probes to gather more detail on menstrual pain, confirm our understanding of participants’ responses, and address any participant confusion.

**Table 1:** Example questions asked by interviewers.

• Tell me about the most recent time you got your period. • How has getting your period affected your life? • How has your life changed after starting your menstrual period? • What are some of the best things about being a young girl in Tanzania? • What challenges does a girl face during her period? • Is there anything you wish you could change about getting your period? • You said that you have abdominal cramps when you are in your period: could you describe it? ○Where in your body do you feel pain? ○When does the pain start? When does pain get the worst? When does pain end? ○What do you do when you get cramps? ○What makes the pain better? ○What makes the pain worse? ○Does the pain keep you from doing anything? ○Does the pain impact your feelings? ○Who taught you how to use medicine for the pain?

#### Analysis

Interviews were transcribed by research assistants in Tanzania, who also completed quality assurance checks on each other’s transcriptions. Transcripts were translated into English twice (once by a research assistant and once by a professional translator) to confirm the accuracy of the translations. Due to having *a priori* theories of interest for a larger study on stress and coping, the qualitative theoretical framework for the overall study was thematic content analysis. Methods from a grounded theory approach, such as line-by-line coding of entire transcripts, were also used to capture any emergent themes and reduce bias; thus, a grounded theory approach was used to describe emergent themes regarding dysmenorrhea, and no *a priori* theoretical stance regarding dysmenorrhea was applied to our analysis [31].

Three researchers (EMC and two research assistants) coded adolescent interviews and adult expert interviews separately using NVivo Version 12 [32]. The code list was developed through an iterative, collaborative process. All interviews were double-coded using a line-by-line descriptive coding strategy. Attention was paid to repeated themes, negative cases, and minor themes. Discrepancies were discussed early in the coding process when the initial code list was being developed (first cycle coding) and again towards the end of the coding process. When examining the subset of codes pertaining to stress and coping, there was initial disagreement in coding for 2.8% of the coded sections for the adolescent interviews and 6% of the coded sections for the adult expert interviews. Discrepancies were resolved using group discussion among the research team. Themes were discussed with Tanzanian research assistants to confirm accuracy.

## Results

**Fig 1:**
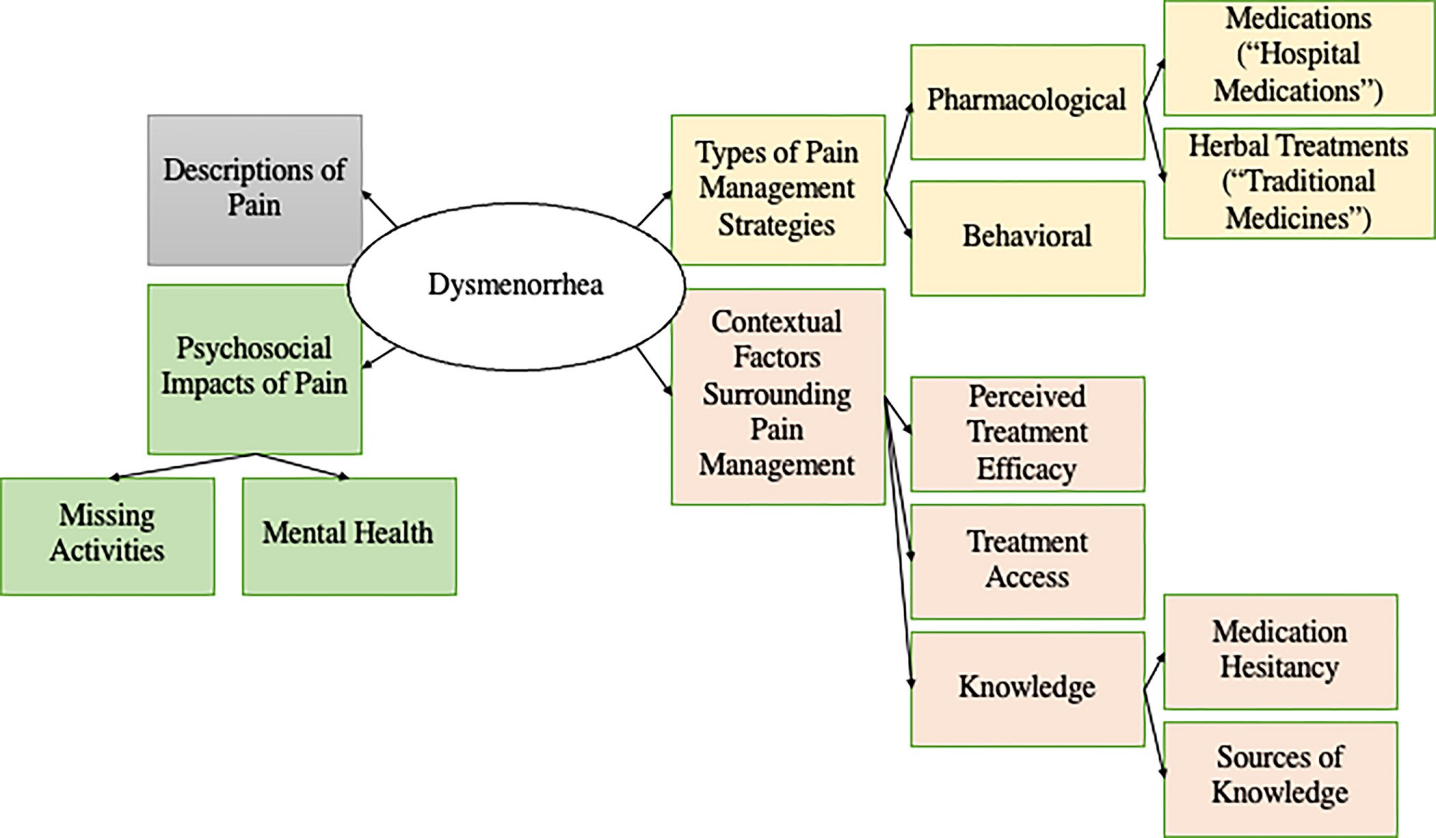
Concept map of themes related to dysmenorrhea in interviews with adolescent girls (n = 10) and adult experts (n = 10) in Moshi, Tanzania.

A concept map of themes related to dysmenorrhea is presented in Fig 1.

### Sample description

Ten adolescent girls and ten adult experts were enrolled. Adolescent participants were between the ages of 14–19 (Table 2). Experts included adults between 30–60 years old, including three NGO staff members (e.g., shelter staff, educators), two teachers, a faith community leader, a pharmacist, two medical doctors, and a mother who had raised adolescent girls. Three local experts were male, including an NGO staff member, teacher, and healthcare provider.

**Table 2:** Adolescent participant demographics, n = 10.

Age	Pregnancy Status	Setting	School Status
14	never pregnant	urban	in school
15	never pregnant	urban	in school
15	never pregnant	rural	in school
15	never pregnant	rural	out of school
17	never pregnant	urban	in school
17	never pregnant	rural	in school
17	never pregnant	urban	out of school
18	previously pregnant	urban	finished secondary school
19	currently pregnant	urban	in school
19	previously pregnant	rural	out of school

### Descriptions of dysmenorrhea

Nine adolescent girls reported experiencing menstrual pain. This included six girls who named dysmenorrhea as a primary challenge in response to open-ended questions about stressors during puberty and three girls who confirmed they had experienced menstrual pain in response to probing from interviewers (e.g., “*Do you experience physical problems before or during your period*?”). One girl did not personally experience menstrual pain.

Interviewer: *What are the bad things or the good things that you went through in the process of growing up*?

Participant: *To start with the bad things*, *I normally go through severe abdominal cramps*. *I get sick*, *and sometimes I don’t go to school*, *and I have to stay at home until I get well*. *Much blood gets out*. *It causes me a lot of discomfort*, *and it is something that really annoys me*–Adolescent E (17, urban setting, in school)

Five girls had severe dysmenorrhea, which can be defined as a high level of pain that 1) causes functional impairment (e.g., being unable to attend school), 2) is not relieved by medications, and/or 3) is accompanied by symptoms such as headaches, fainting, diarrhea, vomiting, or heavy bleeding [33]. Pain severity varied between menstrual periods, across days within a single menstrual period, and within the course of a day.

### Psychosocial impact of dysmenorrhea

Dysmenorrhea and co-occurring symptoms had a negative impact on girls’ mental health and quality of life. Girls reported having to miss school, daily chores, work, physical activity, and social events due to pain. Dysmenorrhea was linked to difficulties concentrating and feelings of annoyance, irritability, anger, fatigue, and sadness. For example, girls reported missing enjoyable activities such as playing with friends, which led to feelings of sadness.

*Another challenge is that I get so much pain that makes me unable to carry on with my work*.–Adolescent J (17, urban area, out of school)

*I feel so bad that I fail to concentrate*, *and my mind cannot function to do anything*.–Adolescent G (17, rural area, in school)

Adolescent girls reported increased irritability and anger due to menstrual pain, which was also observed by adult experts.

Interviewer: *Does it [pain] affect your feelings in any way*?

Participant: *Yes*. *I get angry easily and I cannot hang with someone for more than two minutes because the person irritates me*, *and I can even hit them*.–Adolescent E (17, urban, in school)

*They usually have mood swings*, *which can make it difficult to cope with others*.–Adult Expert (NGO Staff, Male)

### Types of dysmenorrhea management strategies

Pain management strategies reported by girls included pharmacological strategies (including medications and herbs) and behavioral strategies (see Table 3). Behavioral strategies were reported more often than pharmacological strategies. Girls typically combined multiple pain treatments.

**Table 3:** Dysmenorrhea management strategies reported by adolescent girls in Tanzania, *N* = 10.

Pain Treatment Strategy	n	Adolescent Girl Reporting Strategy									
		A	B	C	D^1^	E	F	G	H	I	J
**Pharmacological**											
Paracetamol/acetaminophen	4										
Traditional medicine (herbs)	3										
Non-steroidal anti-inflammatory	1										
Unknown medications^2^	1										
**Behavioral**											
Resting	6										
Drinking water	5										
Physical activity	3										
Heat on abdomen	2										

*Note*. ^*1*^Did not personally experience menstrual pain, but had been instructed to exercise to manage pain. ^2^Adolescent was unaware of medication’s name.

### Types of pharmacological strategies

Medications provided by a doctor or available at a pharmacy were labeled as “hospital medications,” whereas herbal treatments, such as teas, were described as “traditional medicine.” Traditional medicines reported by girls included teas, plants, herbs, and honey.

*It is some kind of herbal medicine which is ground into powder form*, *mixed with honey*, *and I was told to lick it*.–Adolescent E (17, urban area, in school)

Hospital medications taken by girls included the non-NSAID analgesic paracetamol (*n* = 4), the NSAID ibuprofen (*n* = 1), and an unknown medication (adolescent could not recall the name) (*n* = 1). No girls reported taking hormonal contraceptives. Five adult experts in our study recommended medication to treat menstrual pain, including hyoscine butylbromide (i.e., Buscopan, an anticholinergic medication that serves as a gastrointestinal antispasmodic), paracetamol/acetaminophen (a non-NSAID analgesic and antipyretic), NSAIDs, and tramadol (an opioid analgesic). No adolescent participants, however, reported using Buscopan or tramadol. Healthcare providers reported that they preferred Buscopan over other medications, including NSAIDs.

*The first thing we give them are muscle relaxants*, *as the pain is caused by the muscle contractions*.–Adult Expert D (Medical Doctor, Woman)

*The best medicine which I can give a girl*, *which reduces pain quickly is Buscopan*. *Buscopan is better than ibuprofen and paracetamol because it does work very quick to reduce menstrual period pain*.–Adult Expert J (Pharmacist, Woman)

### Type of behavioral strategies

Behavioral strategies used by girls included resting (including lying down and sleeping); increasing fluid intake and drinking hot water; physical activity; and placing hot water on the abdomen.

*I take hot water and put it into a container*, *and I use it to massage my stomach*, *and as I do this the pain subsides*.–Adolescent E (17, urban setting, in school)

Girls were able to rest by getting permission from parents or teachers to miss school or by getting help with chores. Specific physical activities reported by girls included heavy chores (e.g., farming), playing, jumping rope, and other general exercise. Despite the use of physical activity among some girls, other girls had to decrease physical activity during menstruation because of physical symptoms (pain, fatigue, nausea) or worries about leaks due to a lack of menstrual supplies, such as absorbent materials and underwear.

*I find my fellow girls are playing a very good game*, *but I cannot join in because I am in my period and because they are jumping around*, *and I cannot do that*. *It makes me sad*, *and I hate it*.–Adolescent G (17, rural setting, in school)

*Some of them cannot afford the sanitary pads*, *and so they use pieces of cloth*, *and when you use pieces of cloth it is easy for the blood to leak*.–Adolescent E (17, urban setting, in school)

Six girls positively reframed menstrual cramps as a useful sign that menstruation is about to start. This allowed them to prepare by having menstrual supplies available.

### Contextual factors surrounding dysmenorrhea management

#### Perceived treatment efficacy

Reports of treatment efficacy were mixed for hospital medications, herbal treatments, and behavioral strategies, with efficacy for each method ranging from no pain relief to full pain relief. One medical doctor noted that the effectiveness of pain treatments depends on the severity of the pain, and stronger medical treatments are needed for girls who have gynecological disorders such as endometriosis.

*I just put up with the pain*. *Some of the medicines do not help you to feel better*.–Adolescent B (18, urban setting, finished school)

*There is honey medicine that was made for me*, *and I was told to use it*. *I used it for about three months during which I never felt any abdominal cramps… I mean I had no pain at all–*Adolescent E (17, urban setting, in school)

*They [medications] usually help*. *It’s very rare that they fail*. *Mostly it’s with the endometriosis cases*, *where they need an injection first*, *because painkillers do not work with them*.–Adult Expert D (Medical Doctor, Woman)

#### Treatment access

Girls reported receiving herbal treatments from family members or growing medicinal plants at their houses. Adult experts and adolescent girls both agreed that medications are available from healthcare providers and pharmacists at clinics, hospitals, and pharmacies. One of the pharmacists reported that girls must get permission from a parent to get hormonal contraceptives, and the pharmacist would only dispense hormonal contraceptives for pregnancy prevention.

*If she [a girl] is still at home*, *she must get a permission from her parent [to get hormonal contraceptives]*, *because if by any chance a parent realizes she is using family planning without permission it is not right*, *and if she is asking for family planning medicine for another use*, *as a pharmacist I will not allow that*. *It is only for pregnancy prevention*.–Adult Expert J (Pharmacist, Woman)

Adult experts also reported that medication accessibility was limited by costs.

*When a girl is going through severe pain the family cannot afford to take her to the hospital for treatments*.–Adult Expert C (Pastor, Woman).

*In government hospitals*, *they usually announce that they [contraceptive pills] are given out for free*, *but it’s not true*, *because you have to pay for consultation fee*, *so if you do not have money*, *you still won’t be able to access them*.–Adult Expert I (Medical Doctor, Male)

To manage financial costs, one medical provider said some patients had enrolled in a local microinsurance initiative (insurance with low premiums and limited coverage often designed for low-income families) that paid for women’s health visits. Girls did not describe costs related to medications, although they were not specifically asked about financial costs. However, girls did mention that costs were a barrier to accessing menstrual management supplies generally, and when asked what support should be provided to girls during puberty, one adolescent noted that girls should be provided with pads and medicine:

*The first thing is to get the sanitary pads so that they can take care of themselves*. *The second is that*, *to those who get a lot of pain during their period*, *they should get medicine that will be able to relieve abdominal cramps completely*.–Adolescent Girl E (17, urban setting, in school)

#### Treatment knowledge and medication hesitancy

Girls reported selecting pain management strategies based on advice from medical providers and pharmacists (*n* = 6), aunts (*n* = 3), mothers (*n* = 2), sisters (*n* = 1), television/advertisements (*n* = 1), books (*n* = 1), and teachers (*n* = 1). It should be noted that “aunt” can sometimes refer to any female adult in the community. One adolescent noted that a doctor came to her house to provide menstrual education:

Interviewer: *What else did the doctor talk about concerning things like the pain*?

Participant: *That the pain will increase*, *and I should do exercises*, *play*, *and jump the rope*. *And also to drink hot water*. *He said that whenever I am in this condition*, *I shouldn’t run to lie down because the pain would disappear*.*—*Adolescent G (17, rural area, in school)

Two adult experts suggested girls should be educated that menstrual pain is normal; one noted this should be in addition to providing pain treatments.

*They should get painkillers or take a rest*, *and they should be told that this is normal and temporary until when they give birth*.–Adult Expert E (Mother)

Two adult experts indicated that parents are typically not aware that hormonal contraceptive pills can be used to manage menstruation, as contraceptives are only known for preventing pregnancy. No girls reported taking hormonal contraceptives, but they were not specifically asked about their knowledge of contraceptives for menstrual management. One medical doctor reported that medical providers receive limited training regarding dysmenorrhea in college or medical school, and continuing education is rarely available.

Interviewer: *Do doctors and nurses receive training in dysmenorrhea*?

Participant: *Honestly*, *it ends when they are taught in college*. *After that*, *no one bothers about that anymore*. *Having someone come teach continuing medical education is very rare*.–Adult Expert D (Medical Doctor, Woman)

Multiple girls and adult experts either held personal negative beliefs about hospital medicine (e.g., painkillers, hormonal contraceptives) or knew other people, including friends, family members, and teachers, who held such beliefs. This included beliefs that hospital medications are harmful to organs, that medications cause unwanted changes in menstrual patterns, that painkillers lead to harmful levels of tolerance, and that hormonal contraceptives cause infertility. Two adolescents were told by their aunts that medicines are harmful, but they were not given an explanation as to why. One adolescent believed hormonal contraceptives were only appropriate for married women who already had children. Another adolescent’s teacher discouraged the use of medication before first trying physical exercises to reduce menstrual pain. No participants discussed potential negative side effects from herbal treatments.

*Painkillers during your period are not good*, *because they can bring some harm such as repetitive periods*.–Adolescent H (15, rural setting, in school)

*The body will get used to the painkillers*, *and it will reach a point where they won’t be of help anymore even if they will be prescribed for another disease*. *The painkillers are not so good for the kidney due to the chemicals they contain*.–Adult Expert C (Pastor, Woman)

*The teacher answered that medicine is not good in the body*, *because it becomes poison*, *and the abdominal cramps that they get do not require treatment with medicine*.–Adolescent D (15, urban setting, in school)

*The drugs may prevent you from ever getting children again*. *…One of my aunts told me that it is not good to use medicine*, *but she didn’t explain to me why it is not good*, *so I decided that it wasn’t a good thing*. *Because it is just a temporary thing that is passing*, *I just put up with the pain*.–Adolescent I (15, rural setting, out of school)

#### A summary of barriers to dysmenorrhea management

Of the sociocultural factors impacting dysmenorrhea management, several can be defined as barriers because they may make it more difficult for girls to manage dysmenorrhea. This includes 1) beliefs that medications are harmful; 2) lack of knowledge among caregivers about the benefits of hormonal contraceptives for dysmenorrhea management; 3) needing caregiver permission to receive hormonal contraceptives; 4) lack of continuing education for medical providers; 5) costs of medical visits and medications; and 6) lack of access to menstrual supplies such as pads, which makes it difficult to exercise for pain management.

## Discussion

Adolescent girls and adult experts in Tanzania both reported that dysmenorrhea is a disruptive puberty-related stressor. We found that dysmenorrhea limited girls’ ability to fully participate in work and school, which was in agreement with prior research across SSA [7–14]. Although girls used a variety of pharmacological and behavioral strategies to manage pain, the use of first-line medical treatments (NSAIDs and hormonal contraceptives) was not common, and many girls had ongoing menstrual pain despite trying multiple alternatives. Distress and disruptions to school and work caused by menstrual pain are relevant to multiple Sustainable Development Goals (SDGs), including “Good Health and Well-Being,” “Quality Education,” “Gender Equality,” and “Decent Work and Economic Growth” [34]. To achieve progress towards SDGs and enhance SRH and overall well-being among girls in Tanzania, there is a need to improve access to effective and acceptable dysmenorrhea treatments [24] and prioritize cross-sector advocacy (e.g., SRH, education, mental health) to address menstrual pain [34].

### Research on the effectiveness of pain management strategies used by girls in the current study

The dysmenorrhea management strategies reported by girls and adult experts in this study show varying levels of efficacy in prior research. Although healthcare providers in our study recommended Buscopan for dysmenorrhea, there is no evidence that Buscopan is superior to NSAIDs or hormonal contraceptives in treating menstrual pain [35–37]. Likewise, although girls reported using paracetamol, paracetamol is inferior to NSAIDs for the treatment of dysmenorrhea and is only recommended for those who cannot tolerate NSAIDs [38]. Behavioral pain management strategies mentioned by girls included resting, drinking water, engaging in physical activity, and applying heat to the abdomen. Prior research supports the use of applied heat and physical activity for the treatment of mild menstrual pain [39, 40], but there is limited support for increasing fluid intake to alleviate pain. One semi-experimental study among adolescent girls in Iran found that water intake was associated with reduced dysmenorrhea symptoms [41]. However, these results require replication, and it is unknown if water quality and pre-existing dehydration moderate any beneficial impacts of water intake on menstrual pain.

### Barriers to dysmenorrhea management in the context of sexual and reproductive health

Adolescent girls and adult experts reported numerous barriers to dysmenorrhea management, such as medication hesitancy due to limited or inaccurate knowledge about first-line dysmenorrhea treatments and inconsistent access to medications, gynecological care, and menstrual supplies. These barriers to dysmenorrhea management are reflective of a broader lack of resources and stigma surrounding SRH among adolescent girls [42]. For example, adult experts said many families cannot afford the cost of gynecological care, which is important to both overall SRH and dysmenorrhea management. Girls in our study lacked money to purchase menstrual supplies, such as leak-proof pads. This presented a challenge for both managing menstruation overall and using exercise as a dysmenorrhea management strategy. Prior research has shown that negative attitudes and anticipated stigma are barriers to contraceptive use for the purposes of pregnancy prevention; it is likely that these fears extend to the use of hormonal contraceptives for menstrual management [42, 43]. As one pharmacist noted in the current study, girls may be denied hormonal contraceptives if they do not have parental permission or if they seek to use hormonal contraceptives for reasons other than family planning. Thus, stigma around sexual behaviors may impact dysmenorrhea management if it prevents girls from requesting, or being educated about, hormonal contraceptives for managing menstruation [42, 43]. Overall, dysmenorrhea is an important, yet overlooked, aspect of SRH among adolescent girls. Efforts to improve SRH services or reduce stigma around SRH should include the evaluation and treatment of dysmenorrhea.

### Implications for intervention research and clinical practice

Our research shows that dysmenorrhea causes girls significant distress and makes it harder for some girls to attend school, concentrate in school, complete work, and socialize. This provides support for the idea that interventions to diagnose and treat menstrual pain should continue to be tested as a strategy to improve educational outcomes and psychological well-being [14, 44, 45]. Promising interventions are being implemented in SSA to increase access to menstrual education, menstrual products, soap, clean water, and private and safe latrines [46–49]. A growing number of NGO- and research-led interventions in SSA are specifically addressing menstrual pain by incorporating education, deep breathing, and the dispensation of paracetamol [14, 44, 45]. For example, NGOs such as WoMena (in Uganda) and Femme International (in Kenya and Tanzania) are instructing girls to repurpose commonly available household items, such as water bottles or towels/blankets, to use as heat sources for applying heat to the abdomen [14, 50].

To support additional research in this area, we leveraged results from the current study and prior research in SSA to develop potential strategies for tailoring pain management interventions to be more culturally congruent (i.e., accessible and acceptable) among girls in Tanzania (Table 4). It should be noted that these recommendations would require funding, staff training, and community support. Prior to wide-scale implementation, research is needed to evaluate the efficacy and cost-effectiveness of these strategies.

**Table 4:** Potential adaptations that could be tested to increase the cultural congruence of menstrual pain management strategies in Tanzania.

Pain Management Strategy	Strategies for Increasing Cultural Congruence
Heat on the Abdomen	• Train girls to make heating pads from accessible, low-cost materials (bags of rice, stones, and beans; warm water in containers; warmed blankets). • Provide girls with private space and time to apply heat at school/work (menstrual rooms, empty classrooms, and reserved seating areas). • Train teachers on the importance of allowing girls time and privacy to use these resources.
Physical Activity	• Ensure girls have access to menstrual supplies and underwear, as well as private space, water, and time to change and clean supplies.
Increase Water Intake	• More research is needed to examine efficacy. • Improved access to clean water may be needed. Evaluate water availability prior to making suggestions.
Medications	• Collaborative, community-informed interventions are needed to increase patient, provider, and caregiver education about first-line treatments. • To ensure medications are not in conflict with local norms and improve patient-provider trust, community members should be involved in the development of novel medications. • Medications (and food to take with medications) should be provided at no or low cost in menstrual and first-aid kits at home and in schools. School procurement mechanisms may need to be modified to allow for the purchase of medication and food. • Teachers should be trained to recognize dysmenorrhea as a relevant issue and provide medications. • Microinsurance initiatives may be an existing mechanism to expand treatment access. • Continuing education on dysmenorrhea evaluation and treatment should be offered to medical providers.
Traditional Medicine / Herbs	• More research on the efficacy and safety of medicinal plants is required. • If safe and effective, ideal plants would be those that can be cultivated and processed at low cost by girls.
Resting	• Research is needed to adapt cognitive behavioral stress and pain management therapies for use with girls. • This may include training non-mental health providers to teach relaxation skills, among other methods to cope with pain.
Treating Co-Morbid or Causal Factors	• Research is needed to understand endemic factors contributing to menstrual pain that may require alternative treatments and provision of resources, such as sexually transmitted infections, malnutrition and diet, stress, or missed diagnoses of other reproductive and hormonal disorders. • Stress during menstruation may be reduced by providing girls with consistent access to menstrual supplies, social support during menstruation, and menstrual education.

Our findings suggest that to address inaccurate knowledge about first-line medical treatments for dysmenorrhea, there may be a need to work with community members, including local governing bodies and traditional authority structures (e.g., municipal councils, community leaders), institutions (e.g., hospitals, schools), and individuals (e.g., parents, teachers, girls), to develop educational interventions with culturally tailored messaging on the risks and benefits of hormonal contraceptives and NSAIDs for the treatment of dysmenorrhea [51]. Our study shows the particular importance of dispelling myths about the impact of hormonal contraceptives and painkillers on fertility and overall health, as well as providing information about the value of hormonal contraceptives for menstrual management rather than pregnancy prevention. Education should be targeted towards female relatives/caregivers, who are a primary source of information and menstrual resources for girls. Education may also be needed for men, who frequently control family finances or work as teachers. Research from Kenya suggests holding guided discussions including women and their male partners was effective in altering norms around contraceptive use for pregnancy prevention; studies are needed to examine whether family- or caregiver based discussions would be appropriate for providing accurate information on contraceptive use for dysmenorrhea [52].

Girls were often told that menstrual pain was normal. Sharing experiences and providing education on menstrual pain could de-stigmatize menstrual issues, help girls learn to manage pain, and decrease the fear surrounding pain [14]. However, normalizing menstrual pain may also frame pain as untreatable or not worthy of treatment, preventing girls from seeking medical consultation for both menstrual pain and other pelvic pain disorders [53]. One solution is to inform girls that although menstrual pain is common, bothersome or disruptive pain should still be evaluated by a medical provider, and there are a variety of treatment options available for both mild and severe pain.

As with any healthcare decision, the determination of the risks and benefits of dysmenorrhea treatments must be specific to each patient and ideally result in a collaborative discussion between the provider, patient, and caregivers. This is a key tenant of person-centered care, which varies in definition but broadly focuses on allowing patients to participate as informed decision-makers in their own medical care [54, 55]. Person-centered care has shown generally beneficial outcomes and is particularly effective at improving knowledge around SRH [54, 55]. Implementing person-centered care may require significant paradigm shifts in the approach to patient-provider relationships, and continuing education for healthcare providers is needed to improve youth-friendly SRH services in Tanzania [42].

Adult experts reported that many families face financial barriers to accessing medical care, while adolescents reported financial barriers to obtaining menstrual supplies. Financial barriers could be addressed by providing medications, menstrual supplies, and women’s health visits at a reduced cost; increasing the financial resources available to women; or expanding existing microinsurance initiatives, which may be effective in reducing the overall impacts of poverty on women [56].

Although behavioral pain management strategies may be less effective than first-line medications for treating severe pain, behavioral treatments were more accessible and culturally acceptable than medications in our sample. Behavioral strategies can also be used as adjunctive treatments. Cognitive behavior therapy (CBT) for pain coping is one potential behavioral intervention that was not mentioned by girls or experts in our study but has shown efficacy in prior research [57, 58]. Malleable cognitive processes, such as pain catastrophizing, may be involved in menstrual pain [59, 60]. Menstruation is a particularly stressful time for girls in Tanzania, which could further amplify menstrual pain [25, 61]. Stress-management interventions such as CBT may assist girls in coping with these stressors. Furthermore, individuals with menstrual pain have greater pain sensitivity and are at greater risk for developing other chronic pain disorders [17], which highlights the value of pain management skills broadly applicable to chronic pain. CBT has been studied extensively for chronic pain and is feasible and potentially effective for reducing abdominal pain among adolescents [57, 58]. In an open trial of a mind-body intervention for young women with dysmenorrhea, women reported lower menstrual pain and improvements in pain catastrophizing over time [62]. Basic CBT skills, such as deep breathing, activity pacing, stress reduction, and meditation, could be taught as part of menstrual education sessions. For example, research on deep breathing as a key component of pain management strategies is currently being evaluated for acceptability and efficacy in a school-based menstrual health intervention in Tanzania [45]. Additional research is needed to explore the feasibility, acceptability, and efficacy of CBT strategies for menstrual pain, particularly when taught by non-clinicians.

Some girls in our study reported plant-based treatments were effective in reducing pain, which is consistent with research in SSA and other low-resource settings [11, 51, 63]. Although there may be placebo effects, medicinal plants could contain beneficial compounds, and many have demonstrated the ability to influence uterine spasms [51]. In some cases, plants may be grown at home to improve accessibility. However, plants can contain toxic compounds or require specific methods of preparation to be effective and safe [51]. It is important to identify which plants are being used by girls and test them for safety and efficacy. In addition, it was notable that pharmacological treatments were believed to have negative impacts, but herbal treatments were never described as potentially harmful. These findings highlight the need to investigate sources of medical mistrust and work with community members to conduct research on both pharmacological and plant-based medicines to expand safe, effective, and affordable options for girls [64]. This could be accomplished using “reverse pharmacology” methods, which focus on improving the safety and effectiveness of traditional/plant-based medicines already being used by patients [65].

### Limitations

Research is needed to examine if dysmenorrhea in Tanzania is caused or worsened by endemic risk factors, such as a higher rate of sexually transmitted infections (e.g., pelvic inflammatory disease), malnutrition, or chronic stress, as different treatments would be needed to address these conditions. Findings may be biased by the participation of girls who are comfortable discussing menstruation; girls who are uncomfortable discussing menstruation may have greater difficulties obtaining support for dysmenorrhea management. Given the small sample, we were not able to compare participant responses by sociodemographic characteristics. It is unknown whether findings of this qualitative study would be replicated at the population level. Further quantitative research is needed with a larger sample.

### Generalizability

By purposively recruiting diverse participants, both in school and out of school, and from urban and rural areas, we aimed to increase the likelihood that findings are generalizable to mainland Tanzania. Our findings are in congruence with prior research on the prevalence and impacts of dysmenorrhea and coping with pain among girls in Tanzania and other countries in SSA [10, 11, 14, 15]. This provides some evidence of generalizability to adolescent girls in mainland Tanzania. However, it is unknown if specific cultural beliefs about medications are unique to Tanzania or apply to other regions. A lack of consistent measurement strategies for evaluating pain and pain management across settings is a limitation of the existing body of research on dysmenorrhea in SSA. To confirm findings and assess the extent to which Tanzania is representative of SSA, quantitative studies using standardized measures conducted across countries are needed.

## Conclusions

Dysmenorrhea negatively impacts the physical and psychological well-being of girls in Tanzania. Menstrual pain also hinders girls’ ability to participate in school and work. Although first-line treatments have been developed, these medications and other behavioral pain coping strategies may not be culturally relevant due to local beliefs that medications are harmful, limited knowledge of the benefits of hormonal contraceptives for dysmenorrhea, and a lack of access to resources, such as accurate information about medications, money for medications and medical visits, and menstrual management supplies. To improve SRH disparities among girls in SSA, it is necessary to understand the causes of dysmenorrhea and increase access to effective dysmenorrhea treatments that are acceptable to girls and their caregivers across cultural contexts.

## References

[1] Dawood MY. Primary dysmenorrhea: Advances in pathogenesis and management. Obstet Gynecol. 2006;108: 428–441. doi: 10.1097/01.AOG.0000230214.26638.0c 16880317

[2] Harel Z. Dysmenorrhea in adolescents and young adults: From pathophysiology to pharmacological treatments and management strategies. Expert Opin Pharmacother. 2008;9: 2661–2672. doi: 10.1517/14656566.9.15.2661 18803452

[3] Gedefaw G, Wondmieneh A, Getie A, Waltengus F, Demis A, Wang CC. Dysmenorrhea and associated symptoms in Ethiopia: A systematic review and meta-analysis. J Endometr Pelvic Pain Disord. 2022;14: 106–119. doi: 10.1177/22840265221080107

[4] Mukandila AM, Mpueta AN, Mutombo CK, Mulamba JI, Kabamba ET, Mikenji B. Dysmenorrhea in adolescent girls in Kasai Oriental in the Democratic Republic of Congo: prevalence, determinants and attitude. Rev Médicale Gd Lacs. 2014;3: 434–454.

[5] Muluneh AA, Nigussie T seyuom, Gebreslasie KZ, Anteneh KT, Kassa ZY. Prevalence and associated factors of dysmenorrhea among secondary and preparatory school students in Debremarkos town, North-West Ethiopia. BMC Womens Health. 2018;18: 57. doi: 10.1186/s12905-018-0552-x 29699536 PMC5921558

[6] Oni TH, Tshitangano TG. Prevalence of menstrual disorders and its academic impact amongst tshivenda speaking teenagers in rural South Africa. J Hum Ecol. 2015;51: 214–219. doi: 10.1080/09709274.2015.11906915

[7] Pembe AB, Ndolele NT. Dysmenorrhoea and coping strategies among secondary school adolescents in Ilala District, Tanzania. East Afr J Public Health. 2011;8: 232–236. 23120963

[8] Stoilova D, Cai R, Aguilar-Gomez S, Batzer NH, Nyanza EC, Benshaul-Tolonen A. Biological, material and socio-cultural constraints to effective menstrual hygiene management among secondary school students in Tanzania. Sivakami M, editor. PLOS Glob Public Health. 2022;2: e0000110. doi: 10.1371/journal.pgph.0000110 36962274 PMC10021794

[9] Acheampong K, Baffour-Awuah D, Ganu D, Appiah S, Pan X, Kaminga A, et al. Prevalence and predictors of dysmenorrhea, its effect, and coping mechanisms among adolescents in Shai Osudoku District, Ghana. Obstet Gynecol Int. 2019;2019: 1–7. doi: 10.1155/2019/5834159 31236112 PMC6545782

[10] Aziato L, Dedey F, Clegg-Lamptey JNA. The experience of dysmenorrhoea among Ghanaian senior high and university students: pain characteristics and effects. Reprod Health. 2014;11: 58. doi: 10.1186/1742-4755-11-58 25064081 PMC4113597

[11] Buowari OY. Effect of dysmenorrhoea on the quality of life among secondary school girls in Port Harcourt, Nigeria. Savannah J Med Res Pract. 2014;3. Available: https://www.ajol.info/index.php/sjmrp/article/view/106959

[12] Miiro G, Rutakumwa R, Nakiyingi-Miiro J, Nakuya K, Musoke S, Namakula J, et al. Menstrual health and school absenteeism among adolescent girls in Uganda (MENISCUS): a feasibility study. BMC Womens Health. 2018;18: 4. doi: 10.1186/s12905-017-0502-z 29298699 PMC5753466

[13] Nwankwo TO, Aniebue UU, Aniebue PN. Menstrual disorders in adolescent school girls in Enugu, Nigeria. J Pediatr Adolesc Gynecol. 2010;23: 358–363. doi: 10.1016/j.jpag.2010.04.001 21056354

[14] Rubli J. Monitoring & Evaluation and Impact Report 2017: Successes and Lessons Learnt from the Twaweza Program, Femme International. 2017. Available: https://www.femmeinternational.org/wp-content/uploads/2018/09/Femme-International-ME-Report-2017.pdf

[15] Ameade EPK, Amalba A, Mohammed BS. Prevalence of dysmenorrhea among University students in Northern Ghana; its impact and management strategies. BMC Womens Health. 2018;18: 39. doi: 10.1186/s12905-018-0532-1 29433488 PMC5810012

[16] Osonuga A, Ekor M, Odusoga O. Patterns and predictors of management strategies of dysmenorrhea among ghanaian undergraduate students. New Niger J Clin Res. 2018;7: 39. doi: 10.4103/nnjcr.nnjcr_14_18

[17] Iacovides S, Avidon I, Baker FC. What we know about primary dysmenorrhea today: A critical review. Hum Reprod Update. 2015;21: 762–778. doi: 10.1093/humupd/dmv039 26346058

[18] Marjoribanks J, Proctor M, Farquhar C, Sangkomkamhang US, Derks RS. Nonsteroidal anti-inflammatory drugs for primary dysmenorrhoea. In: The Cochrane Collaboration, editor. The Cochrane Database of Systematic Reviews. Chichester, UK: John Wiley & Sons, Ltd; 2003. p. CD001751. doi: 10.1002/14651858.CD001751 14583938

[19] De Sanctis V, Soliman A, Bernasconi S, Bianchin L, Bona G, Bozzola M, et al. Primary dysmenorrhea in adolescents: Prevalence, impact and recent knowledge. Pediatr Endocrinol Rev PER. 2015;13: 512–520. 26841639

[20] Benshaul-Tolonen A, Aguilar-Gomez S, Heller Batzer N, Cai R, Nyanza EC. Period teasing, stigma and knowledge: A survey of adolescent boys and girls in Northern Tanzania. Francis JM, editor. PLOS ONE. 2020;15: e0239914. doi: 10.1371/journal.pone.0239914 33112868 PMC7592731

[21] Cherenack EM, Rubli J, Dow DE, Sikkema KJ. Sexual risk behaviors and menstrual and intravaginal practices among adolescent girls and young women in tanzania: A cross-sectional, school-based study. Int J Sex Health. 2020;32: 394–407. doi: 10.1080/19317611.2020.1821861

[22] Cherenack EM, Sikkema KJ. Puberty- and menstruation-related stressors are associated with depression, anxiety, and reproductive tract infection symptoms among adolescent girls in Tanzania. Int J Behav Med. 2021 [cited 25 Aug 2021]. doi: 10.1007/s12529-021-10005-1 34195917

[23] Guya E, Mayo AW, Kimwaga R. Menstrual hygiene management in secondary schools in Tanzania. Int J Sci Technol. 2014;3: 27–40.

[24] Nkata H, Teixeira R, Barros H. A scoping review on sexual and reproductive health behaviors among Tanzanian adolescents. Public Health Rev. 2019;40: 4. doi: 10.1186/s40985-019-0114-2 31508247 PMC6724376

[25] Sommer M. Ideologies of sexuality, menstruation and risk: Girls’ experiences of puberty and schooling in northern Tanzania. Cult Health Sex. 2009;11: 383–398. doi: 10.1080/13691050902722372 19326264

[26] Sommer M. Where the education system and women’s bodies collide: The social and health impact of girls’ experiences of menstruation and schooling in Tanzania. J Adolesc. 2010;33: 521–529. doi: 10.1016/j.adolescence.2009.03.008 19395018

[27] Sommer M, Ackatia-Armah N, Connolly S, Smiles D. A comparison of the menstruation and education experiences of girls in Tanzania, Ghana, Cambodia and Ethiopia. Comp J Comp Int Educ. 2015;45: 589–609. doi: 10.1080/03057925.2013.871399

[28] Carrillo JE, Carrillo VA, Perez HR, Salas-Lopez D, Natale-Pereira A, Byron AT. Defining and targeting health care access barriers. J Health Care Poor Underserved. 2011;22: 562–575. doi: 10.1353/hpu.2011.0037 21551934

[29] Guest G, Bunce A, Johnson L. How many interviews are enough? An experiment with data saturation and variability. Field Methods. 2006;18: 59–82. doi: 10.1177/1525822X05279903

[30] Lazarus RS, Folkman S. Stress, Appraisal, and Coping. New York: Springer Publishing Company; 1984.

[31] Cho JY, Lee E. Reducing confusion about grounded theory and qualitative content analysis: Similarities and differences. Qual Rep. 2014;19: 1–20.

[32] QSR International Pty Ltd. Nvivo (Version 12). 2018. Available: https://www.qsrinternational.com/nvivo-qualitative-data-analysis-software/home

[33] Andersch B, Milsom I. An epidemiologic study of young women with dysmenorrhea. Am J Obstet Gynecol. 1982;144. doi: 10.1016/0002-9378(82)90433-1 7137249

[34] Sommer M, Torondel B, Hennegan J, Phillips-Howard PA, Mahon T, Motivans A, et al. How addressing menstrual health and hygiene may enable progress across the Sustainable Development Goals. Glob Health Action. 2021;14: 1920315. doi: 10.1080/16549716.2021.1920315 34190034 PMC8253211

[35] de los Santos AR, Zmijanovich R, Pérez Macri S, Martí ML, Di Girolamo G. Antispasmodic/analgesic associations in primary dysmenorrhea double-blind crossover placebo-controlled clinical trial. Int J Clin Pharmacol Res. 2001;21: 21–29. 11708572

[36] Hernández Bueno JA, de la Jara Díaz J, Sedeño Cruz F, Llorens Torres F. [Analgesic-antispasmodic effect and safety of lysine clonixinate and L-hyoscinbutylbromide in the treatment of dysmenorrhea]. Ginecol Obstet Mex. 1998;66: 35–39. 9580220

[37] Janczura M, Kobus-Moryson M, Sip S, Żarowski M, Wareńczak A, Cielecka-Piontek J. Fixed-Dose Combination of NSAIDs and Spasmolytic Agents in the Treatment of Different Types of Pain—A Practical Review. J Clin Med. 2021;10: 3118. doi: 10.3390/jcm10143118 34300284 PMC8306558

[38] Daniels SE, Paredes-Diaz A, An R, Centofanti R, Tajaddini A. Significant, long-lasting pain relief in primary dysmenorrhea with low-dose naproxen sodium compared with acetaminophen: a double-blind, randomized, single-dose, crossover study. Curr Med Res Opin. 2019;35: 2139–2147. doi: 10.1080/03007995.2019.1654987 31397597

[39] Jo J, Lee SH. Heat therapy for primary dysmenorrhea: A systematic review and meta-analysis of its effects on pain relief and quality of life. Sci Rep. 2018;8: 16252. doi: 10.1038/s41598-018-34303-z 30389956 PMC6214933

[40] Nwaezuoke CA, Gbonjubola YT. Aerobic exercise as a non-medicinal option in the management of primary dysmenorrhea: A critical review. Adesh Univ J Med Sci Res. 2022;4: 3–9. doi: 10.25259/AUJMSR_45_2021

[41] Torkan B, Mousavi M, Dehghani S, Hajipour L, Sadeghi N, Ziaei Rad M, et al. The role of water intake in the severity of pain and menstrual distress among females suffering from primary dysmenorrhea: a semi-experimental study. BMC Womens Health. 2021;21: 40. doi: 10.1186/s12905-021-01184-w 33509179 PMC7845092

[42] Mbeba RM, Mkuye MS, Magembe GE, Yotham WL, Mellah AO, Mkuwa SB. Barriers to sexual reproductive health services and rights among young people in Mtwara district, Tanzania: a qualitative study. Pan Afr Med J. 2012;13 Suppl 1: 13. 23467684 PMC3589247

[43] Boamah-Kaali EA, Mevissen FE, Owusu-Agyei S, Enuameh Y, Asante KP, Ruiter RA. A qualitative exploration of factors explaining non-uptake of hormonal contraceptives among adolescent girls in rural Ghana: The adolescent girls’ perspective. Open Access J Contracept. 2021;Volume 12: 173–185. doi: 10.2147/OAJC.S320038 34764703 PMC8577562

[44] Kansiime C, Hytti L, Nalugya R, Nakuya K, Namirembe P, Nakalema S, et al. Menstrual health intervention and school attendance in Uganda (MENISCUS-2): a pilot intervention study. BMJ Open. 2020;10: e031182. doi: 10.1136/bmjopen-2019-031182 32024786 PMC7044877

[45] Okello E, Rubli J, Torondel B, Makata K, Ayieko P, Kapiga S, et al. Co-development and piloting of a menstrual, sexual and reproductive health intervention to improve social and psychological outcomes among secondary schoolgirls in Northern Tanzania: the PASS MHW study protocol. BMJ Open. 2022;12: e054860. doi: 10.1136/bmjopen-2021-054860 35131831 PMC8823075

[46] Montgomery P, Ryus CR, Dolan CS, Dopson S, Scott LM. Sanitary Pad Interventions for Girls’ Education in Ghana: A Pilot Study. PLoS ONE. 2012;7: e48274. doi: 10.1371/journal.pone.0048274 23118968 PMC3485220

[47] Montgomery P, Hennegan J, Dolan C, Wu M, Steinfield L, Scott L. Menstruation and the cycle of poverty: A cluster quasi-randomised control trial of sanitary pad and puberty education provision in Uganda. Montazeri A, editor. PLOS ONE. 2016;11: e0166122. doi: 10.1371/journal.pone.0166122 28002415 PMC5176162

[48] Phillips-Howard PA, Nyothach E, ter Kuile FO, Omoto J, Wang D, Zeh C, et al. Menstrual cups and sanitary pads to reduce school attrition, and sexually transmitted and reproductive tract infections: a cluster randomised controlled feasibility study in rural Western Kenya. BMJ Open. 2016;6: e013229. doi: 10.1136/bmjopen-2016-013229 27881530 PMC5168542

[49] Wilson E, Reeve J, Pitt A. Education. Period. Developing an acceptable and replicable menstrual hygiene intervention. Dev Pract. 2014;24: 63–80. doi: 10.1080/09614524.2014.867305

[50] WoMena. Beyond product distribution: A feasibility study of introducing a menstrual health component into four secondary schools in Buikwe District, Uganda (phase 1). 2020. Available: https://womena.dk/wp-content/uploads/2020/07/Buikwe-report-phase-1.pdf

[51] van Andel T, de Boer HJ, Barnes J, Vandebroek I. Medicinal plants used for menstrual disorders in Latin America, the Caribbean, sub-Saharan Africa, South and Southeast Asia and their uterine properties: A review. J Ethnopharmacol. 2014;155: 992–1000. doi: 10.1016/j.jep.2014.06.049 24975195

[52] Wegs C, Creanga AA, Galavotti C, Wamalwa E. Community Dialogue to Shift Social Norms and Enable Family Planning: An Evaluation of the Family Planning Results Initiative in Kenya. Bhattacharya S, editor. PLOS ONE. 2016;11: e0153907. doi: 10.1371/journal.pone.0153907 27124177 PMC4849797

[53] Scott KD, Hintz EA, Harris TM. “Having pain is normal”: How talk about chronic pelvic and genital pain reflects messages from menarche. Health Commun. 2022;37: 296–306. doi: 10.1080/10410236.2020.1837464 36112920

[54] Diamond-Smith N, Warnock R, Sudhinaraset M. Interventions to improve the person-centered quality of family planning services: A narrative review. Reprod Health. 2018;15: 144. doi: 10.1186/s12978-018-0592-6 30153846 PMC6114885

[55] Rathert C, Wyrwich MD, Boren SA. Patient-Centered Care and Outcomes: A Systematic Review of the Literature. Med Care Res Rev. 2013;70: 351–379. doi: 10.1177/1077558712465774 23169897

[56] Yarumba T, Kazungu I. Micro insurance: A positive intervention to household income and poverty reduction? Experience from Marangu Tanzania. Res J Finance Account. 2014;5: 120–127.

[57] Bonnert M, Olén O, Lalouni M, Hedman-Lagerlöf E, Särnholm J, Serlachius E, et al. Internet-delivered exposure-based cognitive-behavioral therapy for adolescents with functional abdominal pain or functional dyspepsia: A feasibility study. Behav Ther. 2019;50: 177–188. doi: 10.1016/j.beth.2018.05.002 30661558

[58] Ehde DM, Dillworth TM, Turner JA. Cognitive-behavioral therapy for individuals with chronic pain: Efficacy, innovations, and directions for research. Am Psychol. 2014;69: 153–166. doi: 10.1037/a0035747 24547801

[59] Yilmaz B, Sahin N. The effects of a dysmenorrhea support program on university students who had primary dysmenorrhea: A randomized controlled study. J Pediatr Adolesc Gynecol. 2019; S1083318819303778. doi: 10.1016/j.jpag.2019.12.008 31883905

[60] Payne LA, Rapkin AJ, Lung KC, Seidman LC, Zeltzer LK, Tsao JCI. Pain catastrophizing predicts menstrual pain ratings in adolescent girls with chronic pain: Pain catastrophizing and menstrual pain. Pain Med. 2015; n/a-n/a. doi: 10.1111/pme.12869 26218344 PMC4791196

[61] Ju H, Jones M, Mishra G. The prevalence and risk factors of dysmenorrhea. Epidemiol Rev. 2014;36: 104–113. doi: 10.1093/epirev/mxt009 24284871

[62] Payne LA, Seidman LC, Romero T, Sim M-S. An open trial of a mind–body intervention for young women with moderate to severe primary dysmenorrhea. Pain Med. 2020;21: 1385–1392. doi: 10.1093/pm/pnz378 32022890 PMC7372937

[63] Sultana A, Lamatunoor S, Begum M, Qhuddsia QN. Management of Usr-i-Tamth (Menstrual Pain) in Unani (Greco-Islamic) Medicine. J Evid-Based Complement Altern Med. 2017;22: 284–293. doi: 10.1177/2156587215623637 26721552 PMC5871174

[64] Abdullahi A. Trends and challenges of traditional medicine in Africa. Afr J Tradit Complement Altern Med. 2011;8. doi: 10.4314/ajtcam.v8i5S.5 22754064 PMC3252714

[65] Houriet J, Wolfender J-L, Graz B. Selecting the most promising local treatments: retrospective treatment-outcome surveys and reverse pharmacology. Medicinal Plants as Anti-Infectives. Elsevier; 2022. pp. 501–528. doi: 10.1016/B978-0-323-90999-0.00003–3

